# Quo Vadis?

**Published:** 1899-02

**Authors:** T. J. Happel

**Affiliations:** President Tri-State Medical Association


### Quo Vadis?

BY T. J. HAPPEL, A. M., M. D., PRESIDENT TRI-STATE MEDICAL ASSOCIATION.

An article presented as a presidential address usually opens up a wide field for a congratulatory review of the progress of medicine in the few years immediately preceding the date at which it is read, and for painting pictures of a roseate hue of the future possibilities of surgery and gynecology. Any departure from this routine custom may be frowned upon and denounced as "not regular," but men are generally profited more by the mistakes of themselves and their friends than by their successes. A mistake made impresses an honest student of medicine and surgery—as it does workers in all other branches of life—to the extent that the same error is not likely to be committed again. The lesson has been too deeply impressed to be readily forgotten.

An optimist is as dangerous in medicine as a pessimist, if not more so. The optimist, always looking for the good, sees nothing of the bad of his work. The latter is a bad dream, the sooner forgotten the better. His views lead ultimately to experimentations, even to the extent of the sacrifice of human life by the use of new and untried remedies, when old ones are known to accomplish all that can be desired, or rather all that could be reasonably expected.

The pessimist, expecting little, may lose a life from the non-employment of a remedy that might relieve, but does not destroy by the reckless use of those remedies which do not rest on a firm foundation. I trust that thoughts presented on this occasion may occupy a middle ground, rather than bear to either extreme. In an article or two, read on different occasions at medical meetings, it has been my fortune, good or bad, to call down upon my head the opposition of specialists as a whole, but the approval, as a rule, of the general practitioner. Such was not intentional on my part. Nothing that I then wrote was presented in malice toward any, nor intended as a sarcastic review of the tendency of the present day toward specialism in medicine or surgery, but because I felt that the pendulum had swung in one direction too far, and needed something to prevent the recoil from being too great, with a consequent loss of much of the vantage ground that had been gained in the past ten or fifteen years

The proclamation of the discovery of tuberculin by Koch, and the eager grasping of the long-sought remedy for the cure of consumption, both by the profession and the laity, proved to be almost an "apple of Sodom." For a short time it brought discredit on many researches in the direction of serum therapy; nor have we yet fully recovered from the shock of the failure of tuberculin to do what its most ardent and hopeful supporters claimed for it. To some extent, the treatment of diphtheria with the antitoxin prepared in the laboratories, both of this country and Europe, has been discounted by the collapse of the hopes entertained by the enthusiasts in the use of tuberculin. A still heavier impediment has been thrown in the way of theoretic (?) medicine by the issuing of letters patent in this country for the serum manufactured in Germany, when neither there or in France could letters patent be secured. Hence, I take the position that it is well at all times to call a halt in both medicine and surgery, and take our bearing to ascertain "where we are at," and to ask ourselves, "whither are you going?"

In the olden times, in the practice of medicine, every man was a specialist in everything, from the treatment of worms in babies to what was considered in those days capital operations in surgery. A strong foundation was laid in the then sciences of anatomy and physiology, upon which their superstructures were erected. In *these* days the foundation must be more broadly laid by the study in addition of pathology and all its kindred environments, in order to understand scientific medicine and surgery of today. The microscope, in connection with the culture and study of the so-called disease-producing germs, is made to do the greater part of the diagnostic work that was done by a careful study of the patient and his diseased state at the bedside. The clinical history of cases, upon which so much stress was formerly laid, is being pushed to one side, and we are today taught that no diagnosis can be depended upon that is not confirmed by the specialist in his laboratory. I make the statement as broad as above, because the general practitioner cannot be expected to attain to the same skill in the use of the microscope and the various culture-media as the laboratory student or teacher, whose days are spent with his retorts, test-tubes, test solutions, microscopes and X-ray machines, and his diagnosis would be accepted *cum grano salis,* unless he is able to present a report of an expert (?) in each case, confirming the position assumed by him. I, therefore, ask the question *"Quo vadis?"* in this direction? Is clinical medicine to be ignored? Is the student to be taught that there is no need of the bedside study of his case? I say emphatically, "No." When that is to be done, then the general practitioner must disappear from the scene of action in the medical world, or become a mere tool in the hands of some microscopist. I do not hesitate to take the position that Louis, Wood, Flint and others were just as able to diagnosticate diseases in their day and time; that they could be just as positive in regard to the existence of typhoid fever without the outside helps of today, as we are with all the microscopes and culture-media of the present, in the hands of the most expert users of them. Too often the microscope is made to conform in its teachings to the trend of its user. In doubtful cases, as was remarked by a friend of mine, probably now present in this meeting, the decision of a case as to the existence or non-existence of certain sought-for points depended upon the fee paid and the side upon which it was paid. He did not lay this down as a *rule,* but said it occasionally resulted as stated. I would not be considered as reflecting even indirectly upon our laboratory workers. They have done, and are yet doing, much good by their investigations in the domains of medicine and surgery, but it is true, as was remarked by J. Lewis Smith some years ago—before the microscope was used as much as it is now—that we cannot be positive about the presence of pathological germs which were found on the "border-land of the visible." The microscope, the X-ray machine and all other mechanical appliances should be made use of in the examination and diagnosis of cases, medical and surgical, but when they are expected to supply the want of brain in the physician or surgeon, they will prove themselves delusions and snares. The diagnostic ability must be still developed to the highest point, and those mechanic aids should be called in only when in doubt. The fact that the microscope fails to discover the presence of tubercle bacilli in the sputum is no proof that a person's lungs are not diseased. A case, which I was treating and continued to treat till death claimed its victim, was a clear illustration and proof of this fact. Two examinations of the patient's sputum were made, but neither revealed the presence of the bacillus of tuberculosis, and yet the patient filled a consumptive's grave. An able bacteriologist of this State wrote that the results of his examinations were *negative.* Every disease germ has been found by the microscopist to exist in the secretions of healthy individuals. Th Klebs-Loeffler bacillus, the so-called cause of diphtheria, has been found on the fauces of hundreds of school children in Paris, who were in apparent health. And yet we are told that without the aid of the microscope no positive diagnosis of diphtheria can be made. The carefully studied clinical history of any case will enable the competent physician to make the diagnosis of cases coming under his care, which diagnosis may, and should, then be confirmed by the use of the microscope.

The skiagraph is a most excellent adjunct in surgery, but many a life might be lost while waiting for an expert to bring his machine. The teaching of medicine today, then, is, in my opinion, faulty, to the extent that it is laboring to render the general practitioner of medicine and surgery reliant on machines, not on self. The question then, *"Quo vadis?"* is very applicable in this field of teaching. The country practitioner, whose work has done more for the advancement of medicine and surgery than that of his city confrere—that body of the profession, which has furnished a McDowell, a Sims, a Bozeman, and a host of others who have risen to eminence in the profession of medicine and surgery—should not be discouraged by being told that they could not diagnose with any degree of certainty the diseases of everyday life, because they cannot use the microscope or the Roentgen-ray machine. Their work has stood the test of time, and will continue to bear fruit as long as time shall last. The descriptions of typhoid fever cases furnished by Louis and Wood cannot be improved upon by any modern writer, though he may set forth in his report the fact of the presence of the bacillus typhosis, the bacillus of Eberth—a fact utterly unknown to them—and may even still further prove the existence of typhoid fever by Widal's serum test.

All honor to our microscopists and their confreres, who have made clear many doubtful cases; who have been able to ascertain with the wonderful light of this day the presence of foreign bodies, which could not in other ways be located. They deserve honor, glory and shekels or their work—a work which has cost many useful lives in the profession, and where, in Vienna, we have just had strong proof, in the loss of so many valuable lives in the study and culture of the *materies morbi* of the bubonic plague, that those who embark in such work verily take their lives in their own hands.

These new sacrifices upon the altar of medical science should place the profession still higher in the estimation of the world.

Again, *"Quo vadis?"* will apply to the headlong rush into specialism by the sons of specialists and by those who are in no wise related to specialists. Many of the young men of the present day who enter medical schools, attend lectures, not for the purpose of becoming physicians in the broad sense of the term, but for the purpose of preparing to practice some special branch of the healing art. To the attainment or a desired (?) proficiency in their chosen branch or branches, they sacrifice all of the other departments of medicine. If they *barely* pass in all the subjects except their chosen field of labor, they are content, and return home and begin their life's labor with the distinct understanding that they devote special attention to the eye, ear, nose, throat, and genito-urinary organs, etc. It is understood that they are general practitioners for the time being, but as they become able to do so, will devote their time and attention more and more to their specialty, till finally they expect to do no other work. In the olden times such was not the case. Every one went out from college as a general practitioner of medicine and surgery. He devoted his time and attention to this general work—building well on the foundation that he had laid—until he was firmly established in the general and special principles of medicine and surgery. He then began to study himself and his work, finding out in what special direction his talent, if he had any, lay, and from this beginning developed the well-made specialist—a true specialist, because he was thoroughly posted upon the general diseases and conditions, including their treatment, from which the special disease sprung, hence was better adapted to treat and remove the cause of the disease than his brother specialist of today, who believes he was to the "manor born," or who has been graduated from the medical school of the present day a full-fledged specialist in the strictest sense of the term. The specialist who is evolved under the rule of the survival of the fittest, is, in my opinion, more than able to cope with the one who is born new from a medical school. Do not understand me to mean that I would expect the one evolved to proceed with his work without special study and preparation. When the general practitioner discovers his adaptedness for some particular line of work, then he should return to the medical school and devote his time and attention to that line of work. In this way he can become a success in his line.

Again, specialism, I regret to say, is making an effort in some places to break down the Code of Ethics, upon the ground that as they are not general practitioners, they have a right to consult with any one, whether homeopath, eclectic, Indian doctor or "hoo-doo," who may call them, as there is a common ground in their work upon which all may stand. They hold that homeopathy and eclecticism do not apply in the eye, the ear, the nose, and such like *special* fields of labor. As to the truth of this stricture I refer you to the two factions in the State of New York: the one side upholding the Code of Ethics, the other claiming the right to make a new code to suit their own convenience, the leading spirits in this class being mostly those practitioners who are engaged in special work. Another departure from the Code of Ethics along the lines of special work, was referred to by Dr. T. K. Powell, in a paper read at Jackson, Tenn., at the West Tennessee Medical and Surgical Association, viz., the right to contract prices for work in special lines, regardless of services rendered—a thing condemned in the strongest terms when done by the general practitioner. This must not be understood to mean that the specialist should not have, for himself at least, a fee bill, charging a certain price for an operation, for an ovariotomy, a hysterectomy, a cataract operation, or the enucleation of an eye; but that he should not be allowed to say to his patient, "I will cure (?) you, or treat your case for so many dollars, regardless of time or any other contingencies." This procedure smacks too much of quackery, yet it is done by some who stand high in the profession. If the same thing were done by the general practitioner in the beginning of a case of typhoid fever, he would be expelled from the ranks of regular ethical medicine.

Nor do I mean to say that all cases should be charged alike for the same service rendered. There are patients and times when a charge of five dollars in a given case would be more than fifty dollars or even five hundred dollars to another patient under other surroundings. Specialists, as well as the general practitioner, must have, and if *true* men, will have, their charity patients, who must not only be treated free, but must be cared for, fed and clothed whilst being treated. This often falls to the lot of the God-fearing, Christian physician, and specialist also, in all parts of the civilized world.

Again, *"Quo vadis?"* needs an answer in connection with the manufacture and use of so many proprietary medicines of the present day. One manufacturer places upon the market his special preparation of cascara sagrada, backing it with endorsements of well-known medical men, and in a few weeks another manufacturer follows with a similar preparation which he is ready and willing to prove to be better than any other yet placed on the market, and he supports his assertion by a little longer list of names of physicians who testify to the merits of this specific make—each one of the physicians in both cases having been supplied with as many sample bottles as he thought he could use.

We find all kinds of digestive preparations, by different manufacturing establishments, each differing from the other chemically. almost not at all, yet the array of names of reputable physicians certifying to the fact that each one is the best is almost legion. The preparations of pepsin, bismuth and strychnia, of lactopeptin, wine of pepsin, pepsin cordial, etc., could scarcely be enumerated, each of them possessing virtues, but advertised in many cases about as boldly as many of our patent medicines. The myriad of preparations from the phenol group may be cited as another instance of the craze for something new. Each one can be proven by the endorsement of reputable medical men to be the best. Each one has some peculiar property not possessed by its confrere. One will reduce the temperature without depression; another without any excess of diaphoresis; yet another is guaranteed to stimulate whilst the temperature falls; and another especially relieves the pains due to la grippe, etc. The manufacturers would not place these preparations on the market, and could not afford to spend the hundreds of thousands of dollars used annually in supplying the profession with free samples, if there was not a demand for them. The medical world is groping in the dark for a specific remedy for each and every ill that the human flesh is heir to; hence the manufacture. The researches of Koch, Behring, and others in the line of serum therapy, have aroused the commercial spirit in many lesser lights.

Dr. Leartus Conner, in a paper read before the American Academy of Medicine, in Denver, June 4, 1898, speaks of the diseases of the medical profession, and sums up an answer to the next "*Quo vadis?*" as follows: "Among the diseases of the medical profession now definitely recognized and fully described, are: overcrowding; a vast number of incompetents; large numbers of moral degenerates; crowds of pure tradesmen; blatant demagogues; hospitals organized and conducted to the damage of both profession, patient and people; dispensaries and public clinics of the same character; medical colleges organized for the advantage of the few at the expense of the many, and unmindful of advantage to students; medical societies so conducted as to be a by-word among honest persons, and yet continued to advance the financial profit of their leaders; domination by commercial interests of drug manufacturers and proprietors of secret and proprietary medicines; the prey of money-making corporations, as railways, accident insurance companies and sanitariums; the tool of lodges, secret, and other social societies, etc."

This indictment is strongly drawn, yet who can deny that there is something of truth in every count laid down in it? The profession is over crowded, and is becoming more and more so every year, in spite of the increased cost of a medical education and in spite of the rules and regulations prescribed as minimum educational requirements for entrance upon a course of study in a medical college. Although an effort is being made to protect the public through various laws passed in the different States to regulate the practice of medicine, in some cases requiring diplomas from colleges accepted as in good standing by the board constituted under these laws; and in others, by demanding an examination by a body entirely divorced from all college connections and sympathies, still there remains a "horde of incompetents"—incompetent not so much from a want of book learning, as from a want of personal adaptedness to their chosen profession. That there is in the profession a large number of moral degenerates the records of the war just closed prove too conclusively. What appears to be the disgrace of the generation—the blot upon the medical escutcheon—has been the conduct and management of the medical department of the army raised for the invasion of Cuba and Puerto Rico. If *half only* be true that has been published, the medical service rendered has been a disgrace to the civilized world. Nothing that occurred during our Civil War, when the South was deficient in everything, could be compared with the failures made and reported thus far in the conduct of the hospital service of this campaign. In too many cases, the assignments of surgeons and assistants to the different regiments were made without regard to the ability of the applicant, but were decided by the "political pull' brought to bear on the governor of the State. This has been no worse, if as bad, in Tennessee, than in some of the other States. Gutter-snipes, wine-bibbers, and political dead-beats have had the call on men of ability in cases where the politician's Italian hand could be called into play. It, however, does no good to pursue this branch of my subject.

The moral degenerate is seen in the profession by the increasing number of alcoholics, and morphine-habitues in the ranks of the profession. Men who are recognized as reputable practitioners of medicine are found totally unfitted to minister to the sick and suffering because their perceptions are blunted by the long-continued use of alcohol, morphine or cocain. These men should be barred by special statutes from continuing to practice any branch of medicine or surgery. The professional abortionist is not now, as formerly, shunned as a moral leper. The only thing needed in his case is to maintain a semblance at least of respectability as a compliance with what a friend of mine has termed the "eleventh commandment"—"Do not get caught."

This is not as it should be, yet we see in our daily papers advertisements of Dr. A. or B. who will "treat confidentially all ladies who are in trouble." The profession and the laity know that this is an "ad." of the professional abortionist, yet day by day it is seen and read by all men. *"Quo vadis?"*

The spirit of commercialism has been for some years gaining a strong hold upon our profession. The multiplications of public and private hospitals, under whatever name they are called, whether sanitariums, retreats, or what not, together with the dispensaries and public clinics, and the erection of medical colleges—so-called—in different cross-roads towns, and the *unnecessary* increase of the number of those in cities where one or more already were established, is the result of this spirit of commercialism, a greed for gain, or worse still, of the desire to advertise oneself to the world as a professor, for the purpose of catching the innocent and unsuspecting. Alas, too, our medical societies are not always kept free from the blight of the unscrupulous advertiser. A glance at the programs of the meetings of many medical societies and a comparison of the papers proposed to be furnished for the meeting, and those actually read and discussed, and those put in the hands of the secretaries with a reasonable excuse for the absence of the writer, will present a marked contrast. Many whose names appear as such never expected to present any paper, but it was a cheap way to advertise oneself, if a copy of the program could be put in the hands of his local editor, who would no doubt note the fact that Dr. C. was to present a paper on ———— at the approaching meeting of the Dogtown Medical Society.

This side of the picture has been presented to your gaze, not that there is no reverse—thank God, there is!—but that we may profit by our mistakes.

Let us glance for a few minutes only at the other side of the picture. Specialism is needed to the development of medical science.

Without it the wonderful strides that have been made in the study of etiology and pathology in the decade now almost closed, could not and would not have been made.

Without the microscope and the X-ray machine progress would still further have been delayed; without these mechanic helps, and without skilled specialists to use them we could not stand where we now do. Under the stimulus of specialism the practice of medicine is rapidly passing from the domain of empiricism, and being reduced to the basis of a science. A reason for the administration and use of any remedy proposed is now demanded. All this is not to be attained in a day. The work of years is yet before us. Many of the so-called specialties will be relegated to the general practitioner, as has been the ordinary work of the obstetrican. For years, the attention given to the parturient woman demanded a specialist. Now all has changed, and the general practitioner claims the lying-in chamber as much his work as the sick-room of a typhoid patient. Again, the oculist furnishes learned papers at our medical meetings, upon questions of ophthalmology, that belong to the general practitioner; thus recognizing the fact that there, too, the practitioner of medicine is expected to post himself. He is required to be well informed on minor and general surgery. So that year by year the work of the general practitioner is being enlarged. The demand for his services by life insurance companies is forcing him to prepare himself and his office for examination of the heart, lungs and kidneys; so that special fields are again encroached upon along these lines.

The need of the railroads for medical and surgical aid along their lines at points outside the recognized medical centers, compels the general practitioner to equip himself mentally and otherwise for the ordinary, and in some cases extraordinary, surgical operations. As health officers for States, counties and municipalities, his services are daily more and more in demand, so that whilst the ranks of the medical profession in its general and special branches are being crowded, yet new outlets are being made for this excess, and new fields in which they can be employed open up on all sides. We cannot hope to eradicate from the profession the spirit of commercialism; yet its baleful influences can be lessened by a closer adherence to our Code of Ethics.

In the days of the Master the fields were found to have tares growing with the wheat, and the same thing has continued up to the present time, nearly two thousand years. The same advice given in regard to them in those days will apply in our time. We must bear with them; let them severely alone, lest we damage the wheat. The effort being made in a portion of our ranks to break down our Code of Ethics and set up a new code is a tacit agreement that the tares are of more value than the grain. The erring portion of the fraternity of New York City and the State should be left alone to work out their own salvation. Such efforts as were made in Denver to break down the dividing wall and carry the large body of the profession over to that faction cannot be too strongly condemned. It seems to be a case of Mahomet and the mountain. If the mountain will not come to Mahomet, then Mahomet will go to the mountain.

In concluding, I would ask the Tri-State Society to renew its allegiance to our Code, and press forward to the mark and prize of our high calling. The Society has just cause to be proud of the record that is being made by its members in scientific investigations, and whilst the large part of the glory belongs to those who are investigating and reporting upon the hitherto obscure causes of some of our most common maladies, yet we as a Society can shine in their reflected light. But I have occupied enough of your time, and hence, close, thanking you for having had the privilege and honor of serving you for one year as your president.—*Journal of the American Medical Association.*

